# Safe Trajectory Planning for Incremental Robots Based on a Spatiotemporal Variable-Step-Size A* Algorithm

**DOI:** 10.3390/s24113639

**Published:** 2024-06-04

**Authors:** Haonan Hu, Xin Wen, Jiazun Hu, Haiyu Chen, Chenyu Xia, Hui Zhang

**Affiliations:** School of Intelligent Systems Engineering, Sun Yat-sen University, Shenzhen 518107, China; huhn3@mail2.sysu.edu.cn (H.H.);

**Keywords:** mobile robot, trajectory planning, multi-agent robotic systems, incremental robot, collision avoidance

## Abstract

In this paper, a planning method based on the spatiotemporal variable-step-size A* algorithm is proposed to address the problem of safe trajectory planning for incremental, wheeled, mobile robots in complex motion scenarios with multiple robots. After constructing the known conditions, the spatiotemporal variable-step-size A* algorithm is first used to perform a collision-avoiding initial spatiotemporal trajectory search, and a variable time step is utilized to ensure that the robot completes the search at the target speed. Subsequently, the trajectory is instantiated using B-spline curves in a numerical optimization considering constraints to generate the final smooth trajectory. The results of simulation tests in a field-shaped, complex, dynamic scenario show that the proposed trajectory planning method is more applicable, and the results indicate higher efficiency compared to the traditional method in the incremental robot trajectory planning problem.

## 1. Introduction

In the field of robot trajectory planning, there is an unsolved problem: safe and efficient trajectory planning when introducing a new robot into a complex motion scenario without affecting the existing moving robots. The purpose of this paper is to explore how to address this specific and unresolved challenge.

The challenge of this problem is introducing a new robot into a complex motion scenario. This necessitates the consideration of various factors, including the interaction effects between the incremental robot and the existing robots, the representation of the motion states of the different robots, as well as the realization of safe trajectory planning under complex spatiotemporal constraints [1]. Solving this problem is crucial for the operation of multi-agent robotic systems, particularly in robotic network applications.

From the perspective of spatiotemporal constraints, especially when temporal information is considered, unlike trajectory planning, path planning usually focuses only on spatial information, and it is difficult to fully consider the temporal information of robot motion [2]. This leads to path planning methods not adapting to changes in the time dimension and difficulty in avoiding collisions in dynamic motion scenarios, especially when incremental robots are introduced [3,4]. This highlights the challenges of complex spatiotemporal constraints and introducing new robots in dynamic scenarios. The criticality of temporal information constraints further underscores the necessity for in-depth research and innovative solutions to this problem.

### 1.1. Existing Methods

Several studies have used decoupled spatiotemporal planning to accomplish trajectory planning [5]. Decoupled spatiotemporal planning usually separates spatiotemporal information, considering spatial information first and then addressing temporal information [6]. Commonly used spatiotemporal decoupling planning methods include the search method [7], sampling method, and numerical optimization method [8]. For example, Wen, L. [7] used a hybrid A* algorithm for multi-agent trajectory planning based on conflict detection. However, the decoupled spatiotemporal solution severely limits the flexibility and adaptability of the results as the trajectories are tightly correlated in terms of paths and velocities. Although decoupled planning performs reasonably well in some simple scenarios, it is prone to the problem of suboptimal trajectories in complex, dynamic environments [8]. Spatiotemporal decoupling methods cannot easily express the complex spatiotemporal constraints arising from the introduction of incremental robots in dynamic environments; thus, it is difficult to generate trajectories that are both safe and efficient.

Spatiotemporal joint planning methods consider both temporal and spatial information and directly solve for drivable trajectories [9,10]. Traditional spatiotemporal joint planning methods such as the technique developed by Luo, J. [11] use the two-dimensional A* algorithm as the initial path, add the velocity information as the reference trajectory, construct a spatiotemporal corridor based on the reference trajectory, and use segmented Bessel curves for trajectory optimization within the drivable corridor to obtain the final trajectory. Li, J. [12] proposes the use of trapezoidal spatiotemporal corridors to obtain a larger solution space and thus a more optimal solution.

Although the joint spatiotemporal planning approach has a lower solution efficiency due to increased complexity, it has a larger solution space and flexibility and performs better in the multi-agent trajectory planning problem. The method in this paper also belongs to the spatiotemporal joint planning method. However, in the problem studied in this work, the common generation methods of spatiotemporal corridor methods are usually based on a completely sliced initial state, which makes it difficult to generate the solution space of incremental robots without affecting the existing robots [13]. In addition, the conventional spatiotemporal joint planning methods are often based on immutable voxel grids, which complicates the consideration of the motion states of different robots.

Learning-based methods have also been widely used in the field of multi-agent trajectory planning [14], with Reijnen, R. [15] obtaining heuristic values based on reinforcement learning and Sinkar, M. [16] processing dynamic objects based on deep learning models. Although the above methods can effectively generate robot trajectories in simple task scenarios, most of them seldom consider the constraints of motion scenarios and thus cannot easily be applied to the incremental robot trajectory problem in complex scenarios [17].

Advanced techniques such as neural networks and brainstorming optimization have also been reported for path planning. Hills J [18] introduced a method using cellular neural networks (CNNs) for real-time robotic path planning, drawing an analogy between heat conduction and path planning. This method uses the target as a heat source and obstacles as Dirichlet boundary conditions, creating a transient-state heat conduction model to generate temperature fields for optimal path planning. Similarly, Zhong, Y. [19] proposed a neural dynamics approach for optimal path planning, treating the target activity as an energy source propagating through local cell connectivity, ensuring real-time generation of smooth and optimal paths. Zhou, Q. [20] designed a crossover recombination-based global-best brain storm optimization (GBSO) algorithm for UAV path planning, addressing multi-constraint 3D path planning by optimizing a cost function that includes safety, economy, and flyability, using cubic B-spline curves and differential evolution techniques. These advanced techniques offer potential advantages but have yet to be thoroughly integrated with incremental robot trajectory planning in multi-agent systems.

In summary, previous studies have not thoroughly investigated the trajectory planning problem of introducing incremental robots into a complex motion scene with existing intelligent robots in motion, and it is difficult for the existing methods to fully consider the motion speeds of different robots in the motion scene [21,22,23]. Existing methods may not be able to iterate the incremental robot when faced with this problem, which may cause the system to be paralyzed by errors [7], or the problem may not be solved due to the complex spatiotemporal constraints [5], and thus, new methods are needed.

### 1.2. Contributions

This paper will explore an innovative approach to the incremental robot trajectory planning problem described in the introduction. The proposed method, termed Safe Trajectory Planning with Spatiotemporal Variable-Step A* (STP-STVS-A*), involves a spatiotemporal variable-step-size A* algorithm, which is optimized with comprehensive consideration of spatiotemporal constraints to achieve safe and efficient trajectory planning for incremental robots. The primary contributions of this paper are as follows:

Resolution of Incremental Robot Trajectory Planning Problem: This study addresses the challenge of planning trajectories for incremental robots, ensuring that incremental robots can be incorporated into existing systems without affecting existing robots. This resolves a critical issue in managing complex and dynamic environments. The entirety of the paper is dedicated to achieving this objective.

Introduction of the Spatiotemporal Variable-Step-Size A* Algorithm: The spatiotemporal variable-step-size A* algorithm is introduced, which adapts variable step sizes to specific spatiotemporal constraints. This approach improves efficiency and feasibility compared to traditional A* algorithms.

Comprehensive Evaluation and Simulation Analysis: The paper includes extensive simulation analysis comparing the proposed method with existing approaches. The results highlight the effectiveness of the STVS-A* algorithm in addressing the unique challenges of incremental robot trajectory planning in multi-agent systems.

### 1.3. Section Summaries

The remainder of the paper is organized as follows. Section 2 introduces the trajectory planning method, including the method flow; the construction of known conditions, i.e., the establishment of spatiotemporal grid maps; and the computation of the variable time step.

Section 3 introduces the method of calculating the initial spatiotemporal trajectory using the spatiotemporal A* algorithm with a variable time step, including its searching process and node expansion method and the numerical design of the algorithm.

Section 4 introduces the method of instantiating the initial spatiotemporal trajectory, including the B-spline curve fitting method and the numerical optimization method of control points based on constraints.

Section 5 discusses the simulation analysis conducted using the proposed method, presents a comparison with the existing planning methods, and explores the proposed method’s applicability to the incremental robot trajectory planning problem.

Finally, the conclusion and future work are discussed in Section 6.

## 2. Overview

This paper will explore an innovative incremental robot trajectory planning method for solving the problem described in the introduction. The STP-STVS-A* involves a spatiotemporal variable-step-size A* algorithm based on a variable time step, which optimizes the spatiotemporal constraints of incremental and existing robots with comprehensive consideration and achieves safe trajectory planning for incremental robots without affecting existing robots.

### 2.1. Method Flow

**Figure 1:**
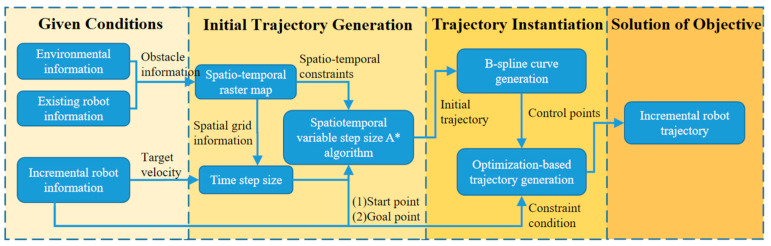
The overall flow of the method.

The overall flow of the STP-STVS-A* is shown in Figure 1.

In the first step, the known conditions are constructed, the environment information and the existing robot information are input, the spatiotemporal grid map is constructed, and its variable time step is calculated according to the incremental robot information.

In the second step, the initial spatiotemporal trajectory is calculated, the incremental robot information is input, the incremental robot time step and spatiotemporal grid map are created, and the search process is conducted using the spatiotemporal A* algorithm with a variable time step. The search process is based on a variable time step to ensure that the incremental robot completes the search at the target speed. The constraint regulation for node feasibility determination is set to ensure non-collision with the trajectory of the subsequent optimization of the space to obtain the initial spatiotemporal trajectory.

In the third step, trajectory instantiation is carried out; the initial spatiotemporal trajectory point is taken as the initial control point of the B-spline curve using B-spline curve fitting; and numerical optimization considering constraints is carried out for the control point to realize the trajectory instantiation and generate the final smooth trajectory.

### 2.2. Spatiotemporal Grid Map Construction

When constructing known conditions, the first step is to construct the spatiotemporal grid map. The traditional two-dimensional XY grid map can only represent spatial information and cannot directly cover the time dimension, so it is necessary to reconstruct the map by adding the time T-dimension information as the third dimension to construct the X-Y-T three-dimensional spatiotemporal grid map [24].

Given that the robots in the scenario are not driven by human beings, there is no need to consider semantic information. Considering the complexity of the situation, in accordance with the research by Li, B. [25], the Cartesian coordinate system is employed to maintain consistency in scenario description, thereby ensuring completeness of expression and method applicability.

**Figure 2:**
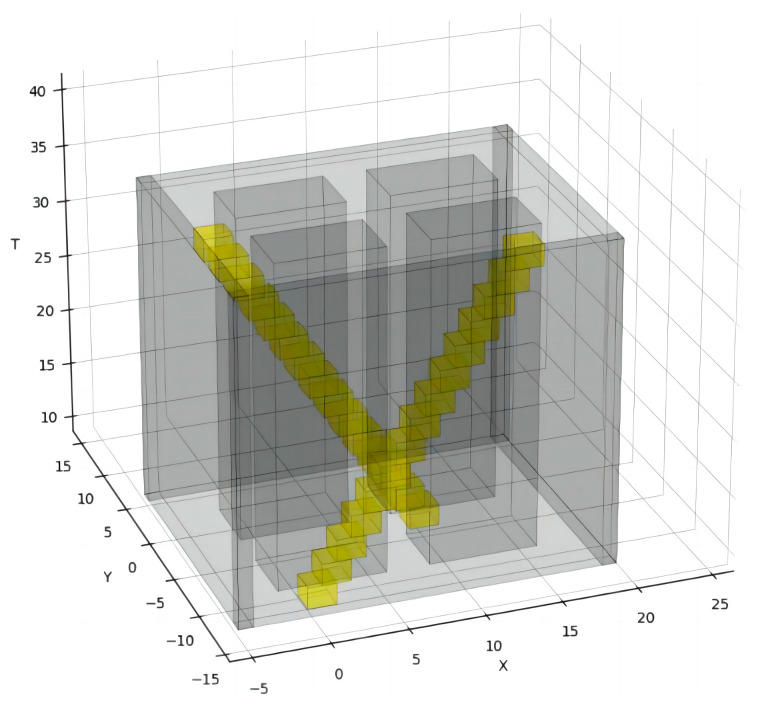
X-Y-T spatiotemporal grid map.

The X-Y-T spatiotemporal grid map is shown in Figure 2. To ensure that multiple robots do not collide, the obstacle information needs to be represented in a 3D spatiotemporal grid map, where the spatiotemporal trajectory of the existing robots is represented as dynamic obstacles visually identifiable in the grid map as regions of XY positional states at various discrete time points [9]. The spatiotemporal trajectory of the existing robots is constructed as described in Section 2.4. Dynamic obstacles, depicted in yellow in the figure, are known and predictable, while static obstacles, shown in gray, are represented as XY regions parallel along the time axis T. Using 3D spatiotemporal grid maps to represent obstacles can clearly represent the positional relationship and relative motion of different robots and obstacles during the time period.

### 2.3. Variable Time Step Representation

The next step in constructing the known conditions requires the calculation of the variable time step of the robots. The traditional algorithm’s time node expansion method is based on an immutable time step for expansion, and it is difficult to express different speed states for the immutable time step, which cannot easily be applied to scenarios involving multiple robots running at different speeds [26].

**Figure 3:**
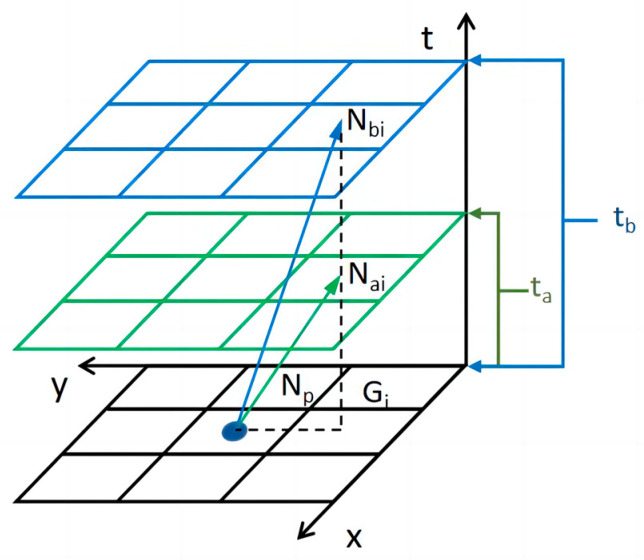
Illustration of variable time step lengths for different robots.

The variable time step method proposed in this paper determines the sampling time step based on the target speed of the robot, and the nodes are expanded for each robot based on the corresponding time step, which is used to express the scene of different target speeds of intelligent running. The variable time step lengths of different robots are shown in Figure 3.

The speed of robots can be expressed as follows:

(1) $$v_{a}=\bar{N_{p}G_{i}}/\bar{N_{ai}G_{i}}=\bar{N_{p}G_{i}}/t_{a}=d_{xy}/t_{a}\;v_{b}=\bar{N_{p}G_{i}}/\bar{N_{bi}G_{i}}=\bar{N_{p}G_{i}}/t_{b}=d_{xy}/t_{b}$$

where $v_{a}$ is the speed of the robot within the time step $t_{a}$, $v_{b}$ is the speed of robot *b* within the time step $t_{b}$, and $d_{xy}$ is the spatial grid step.

Since the spatial grid step is the same, i.e., the spatial position change is the same, the velocity ratio of the robot can be expressed as follows:

(2) $$v_{a}/v_{b}=t_{b}/t_{a}$$

Accordingly, the time steps of the robots can be calculated from the target speed and the spatial grid step as follows:

(3) $$t_{a}=\bar{N_{p}G_{i}}/v_{a}=d_{xy}/v_{a}\;t_{b}=\bar{N_{p}G_{i}}/v_{b}=d_{xy}/v_{b}$$

**Figure 4:**
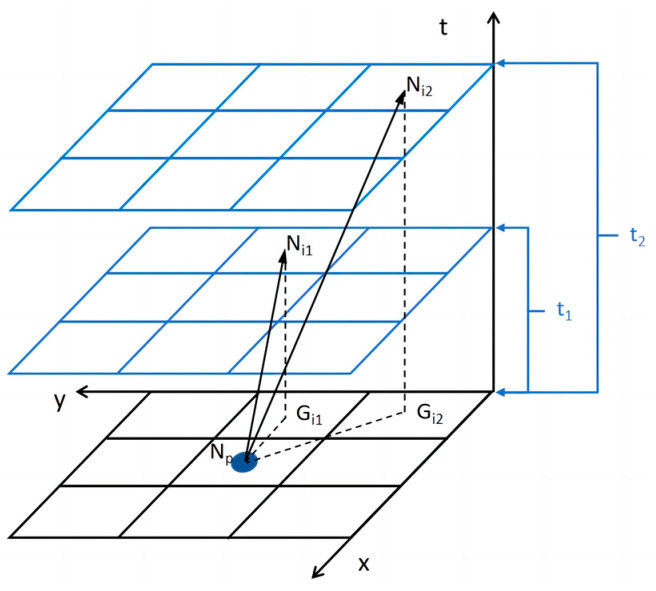
Illustration of variable time step lengths for the same robot.

The velocity maintenance of the same robot also requires the use of a variable time step. The node expansion process with a 45° diagonal expansion in the spatial grid will lead to an inconsistent spatial step. To maintain the same speed, diagonal expansion of the time step can be carried out via horizontal and vertical expansion $\sqrt{2}$ times. The same robot’s variable time step process is shown in Figure 4.

The speed of robots can be expressed as follows:

(4) $$v_{i1}=\bar{N_{p}G_{i1}}/\bar{N_{i1}G_{i1}}=\bar{N_{p}G_{i1}}/t_{1}=d_{xy}/t_{1}\;v_{i2}=\bar{N_{p}G_{i2}}/\bar{N_{i2}G_{i2}}=\bar{N_{p}G_{i2}}/t_{2}=d_{xy}/t_{2}$$

where $v_{i1}$ is the speed of the same robot when traveling horizontally and vertically in space, $v_{i2}$ is the speed when traveling diagonally in space, $t_{1}$ is the time when the same robot travels horizontally and vertically in space, and $t_{2}$ is the time when the same robot travels diagonally in space.

Therefore, the time step of oblique expansion for the same robot while keeping the same target speed can be expressed as follows:

(5) $$t_{2}=\bar{N_{p}G_{i2}}v_{i1}t_{1}/\bar{N_{p}G_{i1}}v_{i2}=\sqrt{2}t_{1}$$

Using the node expansion method with a variable time step can ensure that different robots carry out the search process of the initial spatiotemporal trajectory with the determined target speed and maximize the speed of the final trajectory to be stabilized at the target speed.

### 2.4. Known Condition Construction

An incremental robot is defined as a robot that enters the motion scene whose starting point, end point, and target speed are given in the problem, and its spatiotemporal trajectory needs to be solved to safely move from the starting point to the end point. An existing robot is defined as a robot that has been moving in the scene; that is, its starting point, end point, target speed, and spatiotemporal trajectory are all known. The starting point, end point, and target speed are set conditions. The spatiotemporal trajectory of the existing robots is necessary for solving this problem as it is a given condition of the incremental robot trajectory calculation.

The spatiotemporal trajectory of existing robots is obtained by taking each existing robot as an incremental robot entering the scene at its previous starting point and conducting trajectory calculation through the method proposed in this paper. When the first robot enters the scene, there is no existing robot. Most existing robots enter the scene at different times, so there is a natural iterability between different existing robots. If existing robots enter the scene at the same time, the priority is randomly assigned for trajectory calculation. It is feasible to calculate the trajectory of existing robots iteratively by using this method as a known condition. The spatiotemporal trajectory of all existing robots is obtained by iterating the trajectory of different existing robots, and the trajectory calculation of the incremental robot entering the scene in the problem is carried out and is considered known information.

According to the above calculations, the map information, the existing robot information, the incremental robot time step size, and other known conditions are constructed.

## 3. Initial Spatiotemporal Trajectory Calculation

The initial spatiotemporal trajectory is the control point reference trajectory for subsequent trajectory instantiation, and the reasonableness of the initial spatiotemporal trajectory directly affects the performance and safety of the final trajectory result.

In this paper, a spatiotemporal variable-step-size A* algorithm is proposed for computing the initial spatiotemporal trajectory of incremental robots. The innovation of the algorithm is its new spatiotemporal child node searching method based on a variable time step to ensure that the robot completes the search at the target speed, which improves the practicality and applicability of the method. A new cost function and heuristic function were also designed to reduce the number of search calculations and improve the accuracy of the results.

In this work, the spatiotemporal 3D A* algorithm was selected for improvement. The A* algorithm is a commonly used search algorithm based on the principle of heuristic search, which means that instead of blindly exploring every possible path, the search is guided by some heuristic information, thus finding the best path more efficiently. The flow of the A* algorithm is described in detail in Section 3.3.

In general, the A-star algorithm finds the best path by evaluating the actual cost of nodes and heuristic estimates, and the algorithm is executed efficiently and reliably [27].

Since it is computationally inefficient to optimize the motion constraints in the initial spatiotemporal trajectory computation stage [28], and the motion constraints considered in the initial spatiotemporal trajectory have less influence on the final trajectory after instantiation, motion optimization is carried out in the trajectory instantiation step, and the algorithm is chosen to be improved without considering the kinematic model.

The process of calculating the initial spatiotemporal trajectory is as follows: Firstly, the search is started according to the constructed spatiotemporal grid map with the robot time step. The process of expanding the spatiotemporal nodes is carried out according to set instructions, and a series of constraints are considered for the node feasibility determination in the process. Then, the feasible nodes are evaluated through the calculation of heuristic and surrogate values until the target point is reached and the overall initial spatiotemporal trajectory is obtained. The expansion process is based on a variable time step to ensure that the incremental robot completes the search at the target speed. The search process is described in detail in Section 3.3.

### 3.1. Spatiotemporal Node Expansion and Feasibility Determination

**Figure 5:**
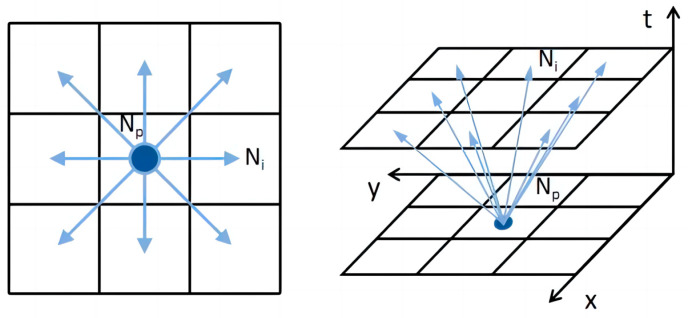
Spatiotemporal node expansion approach.

The search process of the algorithm requires spatiotemporal node expansion. The node state space of the traditional A* algorithm is (x, y) [4]. The X-Y-T three-dimensional spatiotemporal grid map used in this study increases the time dimension information and the corresponding velocity information, so the corresponding spatiotemporal variable-step-size A* algorithm state space is (x, y, t); the first two items are the same as the node state space of the traditional A* algorithm, and the third item is the time node where the current position is located. The spatiotemporal node expansion approach is shown in Figure 5.

The node expansion method based on the state space of spatiotemporal nodes adopted in this study involves expanding along the time axis T with a sampling time step, starting from the parent node, and the spatiotemporal expansion process of the child nodes is expressed as follows:

(6) $$\left\{\begin{array}{l} x_{i}=x_{p}+v_{goal}d_{t}\cos\theta_{i}\\ y_{i}=y_{p}+v_{goal}d_{t}\sin\theta_{i}\\ t_{i}=t_{p}+d_{t} \end{array}\right.$$

where $d_{t}$ is the time step of the same robot, $\theta_{i}$ is the discrete forward direction angle, and $v_{goal}$ is the target speed of the robot.

The time step length $d_{t}$ of the same intelligent robot is given as follows:

(7) $$d_{t}=\left\{\begin{array}{l} d_{xy}/v_{goal},\theta_{i}\in \left\{0,\pi /2,\pi ,3\pi /2\right\}\\ \sqrt{2}d_{xy}/v_{goal},\theta_{i}\in \left\{\pi /4,3\pi /4,5\pi /4,7\pi /4\right\} \end{array}\right.$$

where $d_{xy}$ is the spatial grid step.

Feasibility of the expanded nodes must also be determined [29].

The first is collision determination, which requires the child node to be split based on the minimum time step to obtain a series of transition nodes, and the child node can be considered safe only if neither the child node nor the transition node collides with the obstacle. In addition to collision detection, the child node and its transition nodes need to satisfy the scene boundary constraints and instantiation margin constraints.

The scene boundary constraint is used to ensure that the robot proceeds within the spatial range limited by the scene and does not go beyond the motion scene [30]. Its representation is as follows:

(8) $$\left\{\begin{array}{l} x_{\;}^{lowers}\leq x_{i}\leq x_{\;}^{upper},i=1,\cdots ,N_{c}\\ y_{\;}^{lower}\leq y_{i}\leq y_{\;}^{upper},i=1,\cdots ,N_{c} \end{array}\right.$$

where $x^{upper},x^{lower},y^{upper},y^{lower}$ are the upper and lower boundaries of the scene in the *x*-axis direction and the upper and lower boundaries of the scene in the *y* direction, respectively.

The instantiation margin constraint is used to ensure a certain spatiotemporal margin as the optimization space and error space in the subsequent trajectory instantiation step. And it ensures that the trajectories will not collide, which is expressed as follows:

(9) $$\left\{\begin{array}{l} x_{b}^{lower}+I_{x}\leq x_{i}<x_{b}^{upper}-I_{x}\\ y_{b}^{lower}+I_{y}\leq y_{i}<y_{b}^{upper}-I_{y}\\ t_{b}^{lower}+I_{t}\leq t_{i}<t_{b}^{upper}-I_{t} \end{array}\right.$$

where $x_{b}^{upper},x_{b}^{lower},y_{b}^{upper},y_{b}^{lower},t_{b}^{upper},t_{b}^{lower}$ are the upper and lower boundaries of the obstacles in the x,y,t directions, and $I_{x},I_{y},I_{t}$ are the instantiation margins in the *x*, *y*, *t* directions, respectively.

The instantiation margins include the B-spline fitting margin, control point optimization margin, speed control error margin, and robot geometry margin, and the instantiation margins $I_{x},I_{y},I_{t}$ in the three directions of *x*, *y*, *t* can be expressed as follows:

(10) $$\left\{\begin{array}{l} I_{x}=w_{bx}e_{b}+w_{hx}e_{h}+w_{vx}e_{v}+e_{t}\\ I_{y}=w_{by}e_{b}+w_{hy}e_{h}+w_{vy}e_{v}+e_{t}\\ I_{t}=w_{bt}e_{b}+w_{vt}e_{v}+e_{t} \end{array}\right.$$

where $e_{b}$ is the B-spline fitting margin, and $w_{bx},w_{by},w_{bt}$ are its coefficients in the *x*, *y*, *t* directions, respectively; $e_{h}$ is the control point optimization margin, and $w_{hx},w_{hy}$ are its coefficients in the *x*, *y* directions, respectively; $e_{v}$ is the speed control error margin, and $w_{vx},w_{vy},w_{vt}$ are its coefficients in the *x*, *y*, *t* directions, respectively; and $e_{t}$ is the robot geometry margin.

**Figure 6:**
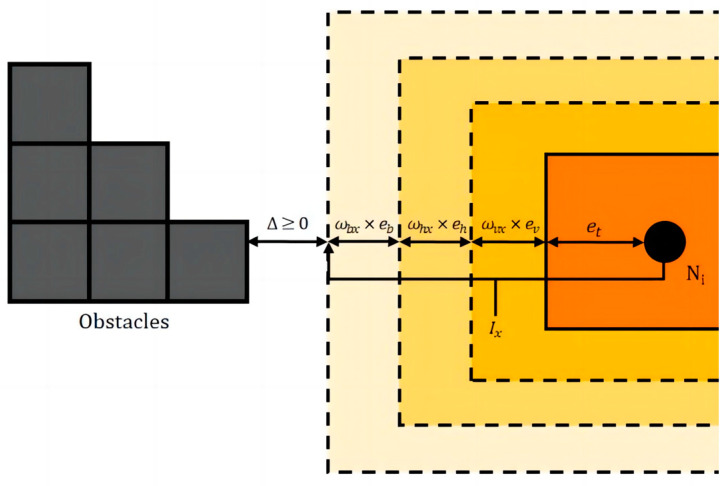
Example of instantiated margin.

The instantiation margin in the *x*-axis direction is shown in Figure 6.

The speed control error margin is used to ensure a certain control error of the robot to avoid the trajectory due to small errors in the control link, thus affecting the safe operation of the robot. It can be expressed as follows:

(11) $$e_{v}=v_{goal}m_{max}d_{t}$$

where $v_{goal}$ is the target speed, and $m_{max}$ is the maximum percentage of control error allowed.

The geometric margin of the robot is used to consider the physical shape of the robot in operation, and half of the maximum value of the length and width of the robot is selected as the safe collision avoidance condition, which can be expressed as follows:

(12) $$e_{t}=\max\left(l_{a},l_{b}\right)/2$$

where $l_{a},l_{b}$ are the length and width of the robot, respectively.

The B-spline fitting margin $e_{b}$ is used to avoid the trajectory boundary after B-spline curve fitting exceeds the safety range, and the control point optimization margin $e_{h}$ is used to ensure that the control points change range in numerical optimization. These two constraints are related to the subsequent instantiation, which is shown in Section 4.1.

The next step of cost evaluation can be performed only after both the child nodes and transition nodes satisfy the constraints and collision detection. Using the improved node expansion approach, the computation of robot trajectories with different target velocities can be achieved while ensuring safety and no collision.

### 3.2. Algorithm Numerical Design

The numerical design of the spatiotemporal variable-step-size A* algorithm mainly consists of two parts: the heuristic function and the cost function.

The spatiotemporal node heuristic function is used to guide the algorithm to search in the direction close to the target point. The initial spatiotemporal trajectory obtained using the spatiotemporal variable-step-size A* algorithm is a trajectory based on the direct connection of the grid endpoints. Information other than the distance from the current point to the target point has little effect on the heuristic search process. The proposed method uses spatiotemporal Euclidean distance as a heuristic function to reduce the amount of computation for a complex, dynamic scene [21]. The child nodes $N_{i}\left(x_{i},y_{i},t_{i}\right)$ of the heuristic function $H_{i}$ can be expressed as follows:

(13) $$\begin{array}{c} H_{i}=w_{H1}\sqrt{{\left(x_{\mathrm{goal}}-x_{i}\right)}^{2}+{\left(y_{\mathrm{goal}}-y_{i}\right)}^{2}}+w_{H2}\left(t_{\mathrm{goal}}-t_{i}\right) \end{array}$$

where the first term is the planar Euclidean distance heuristic term; $x_{\mathrm{goal}}$ and $y_{\mathrm{goal}}$ are the *x* and *y* coordinates of the target point, respectively; the second term is the temporal distance heuristic term; $t_{\mathrm{goal}}$ is the relative time coordinate of the target node; and $w_{H1}$ and $w_{H2}$ are the weights of the corresponding heuristic terms, respectively.

The cost function is used to calculate the cumulative cost from the search start node to the current node to evaluate the optimal expansion node. The total cost $G_{i}$ is composed of the cost $G_{p}$ of the parent node $N_{p}$ and the transition cost between the parent node $N_{p}$ and the child node $N_{i}$, which can be expressed as follows:

(14) $$G_{i}=G_{p}+w_{g1}E_{i}+w_{g2}E_{s}$$

where $G_{p}$ is the cost of the parent node, $E_{i}$ is the cost of the speed deviation of the child node, and $E_{s}$ is the cost of the security risk of the child node.

The cost of the speed deviation of the child node can be expressed as follows:

(15) $$E_{i}=\left|v_{i}-v_{goal}\right|$$

where $v_{i}$ is the current speed of the child node, and $v_{goal}$ is the target speed of the robot.

Wang M Q’s research [3] was referred to in this study to establish the risk field in the spatiotemporal grid map and improve the security and accuracy of the spatiotemporal variable-step-size A* algorithm. To reduce unnecessary computation in complex scenes, the node cost function only considers the risk cost of static obstacles. The risk field of a point $\left(x_{i},y_{i}\right)$ in the field can be expressed as follows:

(16) $$P\left(x,y\right)=\exp\left[-\frac{1}{2}\left(\frac{{\left(x_{i}-u_{x}\right)}^{2}}{\sigma_{xg}^{{\;}^{2}}}+\frac{{\left(y_{i}-u_{y}\right)}^{2}}{\sigma_{yg}^{{\;}^{2}}}\right)\right]$$

where $u_{x}$, $u_{y}$ are the *x* and *y* coordinates of the obstacle risk source center; $\sigma_{xg}^{\;},\sigma_{yg}^{\;}$ are the distribution factors of obstacles in the *x* and *y* directions, respectively.

Therefore, the static risk field formed by the stationary object O $\left(x_{o},y_{o}\right)$ in the road at its surrounding point $\left(x,y\right)$ can be expressed as follows:

(17) $$\left\{\begin{array}{l} E_{s}=P\left(x,y\right)\frac{r}{\left|r\right|}\\ r=\left(x-x_{0},y-ys_{0}\right) \end{array}\right.$$

where $P\left(x,y\right)$ is the risk distribution model of the robot at the point $\left(x,y\right)$, and $\frac{r}{\left|r\right|}$ is the unit vector of the direction of the static field strength.

### 3.3. Spatiotemporal Variable-Step-Size A* Algorithm Flow

The A* algorithm is structured as follows:

Initially, a grid map is established, with the current incremental robot’s starting position serving as the search starting node, denoted as S. A variable time step is then computed based on the robot’s target speed, and spatiotemporal nodes are expanded accordingly. The starting node, S, is enlisted in a roster of nodes earmarked for consideration, with its initial G value set to signify the actual cost from the starting point to the current node. Subsequently, nodes closest to the starting point are iteratively selected from this roster.

During the expansion process, collision detection and various constraints are taken into account. Feasibility assessments are conducted concerning both child nodes and their transitional counterparts. Additionally, the heuristic function and cost function are leveraged to gauge the cost of nodes, aiding in the quest for the optimal expansion node.

For each chosen node, its H value, representing a heuristic estimation of the anticipated cost from the current node to the target node, is computed, and its F value, the sum of G and H, is updated. This computation aids in evaluating the prospective value of the current node as an intermediary.

Subsequently, the neighbors of the current node are inspected, and their G and H values are computed. If a neighbor node is absent from the roster of nodes under consideration, it is appended, and its G and H values are adjusted accordingly. Conversely, if the node already exists in the roster, its freshly calculated G value is compared with the existing one, with the smaller value retained.

This iterative process persists until either the target node is reached or the roster of nodes under consideration becomes empty. Attainment of the target node signifies the discovery of the optimal path. Conversely, if the roster becomes depleted without encountering the target node, it indicates the absence of a feasible path from the starting point to the target.

**Figure 7:**
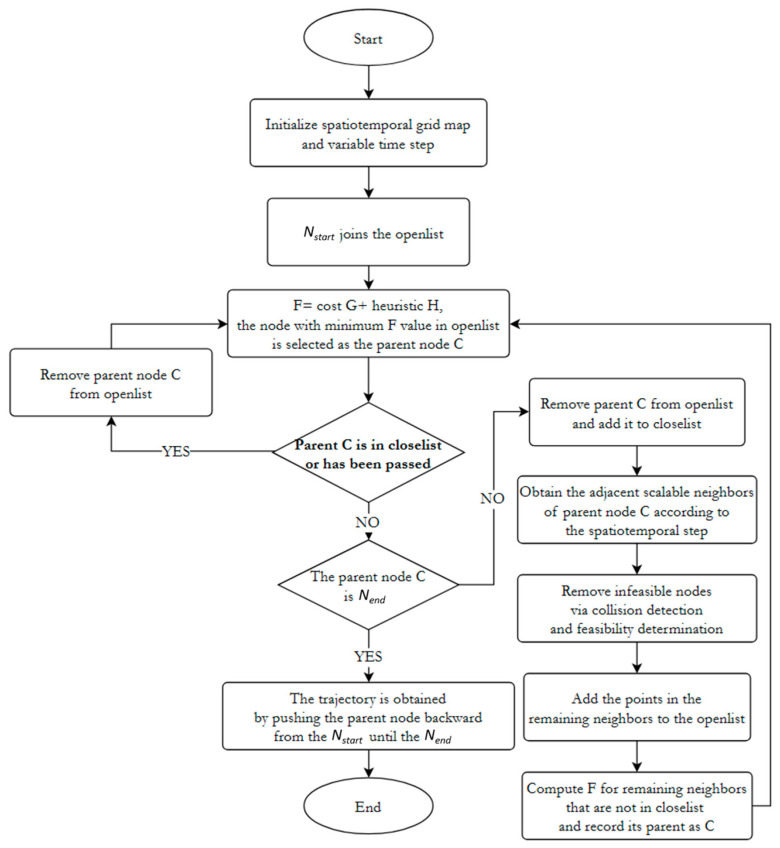
Search flow chart of spatiotemporal variable-step-size A* algorithm.

Throughout the expansion process, the viability of child nodes and their transitional counterparts is assessed, while heuristic and cost functions are employed to evaluate node costs, culminating in the identification of the optimal expansion node. This process continues until the search incorporates the target node, thus concluding the search procedure of the spatiotemporal variable-step-size A* algorithm. The ideal path is obtained at the end by backtracking. Consequently, the initial spatiotemporal trajectory, delineated by discrete grid nodes, is also derived. The detailed search flow is illustrated in Figure 7.

## 4. Spatiotemporal Trajectory Instantiation

The initial spatiotemporal trajectory derived from the spatiotemporal A* algorithm is based on the direct connection of the 3D spatiotemporal grid endpoints, which is jagged, does not conform to the kinematics of the wheeled mobile robot, and fails to meet the control requirements of the mobile robot. To obtain a smooth, safe, and feasible trajectory, it is necessary to instantiate the spatiotemporal trajectory.

Curve fitting can be used to instantiate the initial spatiotemporal trajectory. Compared with other curves, the B-spline curve has the advantages of convex envelope characteristics, local adjustment characteristics, and hodograph characteristics, so it was chosen for instantiation.

The instantiation process is as follows: the initial spatiotemporal trajectory node is taken as the control point; the B-spline curve is used for fitting; all kinds of constraints are comprehensively considered; the objective function is set; the control point is taken as the variable for optimization and solving; and then the final spatiotemporal trajectory is obtained.

### 4.1. B-Spline-Based Instantiation

The B-spline curve is controlled by three factors: the control point, order, and basis function, and the general expression of the B-spline can be written as follows:

(18) $$p\left(t\right)=\sum_{i=0}^{N}Q_{i}B_{i,k}\left(t\right).$$

where $Q_{i}$ is the control point, k is the order, and $B_{i,k}\left(t\right)$ is the basis function, which can be derived from the Cox–de Boor formula [31].

We associate the spatial coordinates of each 3D node in the initial spatiotemporal trajectory with the temporal coordinates $t_{i},i\in \left[k,M-k\right]$ and normalize the time to $s\left(t\right)=\left(t-t_{i}\right)/dt,\;t\in \left[t_{i},t_{i+1}\right]$.

According to Zhang T’s research [32], the formulation of the B-spline can be expressed in terms of a matrix function as follows:

(19) $$p\left(s\left(t\right)\right)=s\left(st\right)M_{k+1}Q_{i},\;s\left(t\right)=\left[1s\left(t\right)s^{2}\left(t\right)\cdots s^{k}\left(t\right)\right],\;Q_{i}={\left[Q_{i-k}Q_{i-k+1}Q_{i-k+2}\cdots Q_{i}\right]}^{T},\;M_{k+1}=\left[\begin{array}{cccc} m_{0,0} & m_{0,1} & \cdot \cdot & m_{0,k}\\ m_{1,0} & m_{1,1} & \cdot \cdot & m_{1,k}\\ \vdots & \vdots & \cdot \cdot & \vdots \\ m_{k,0} & m_{k,1} & \cdot \cdot & m_{k,k} \end{array}\right]\;m_{i,j}=\frac{1}{k!}C_{k}^{k-i}\sum_{s=j}^{k}\;{\left(-1\right)}^{s-j}\times C_{k+1}^{s-j}{\left(k-s\right)}^{k-i},\;C_{n}^{i}=\frac{n!}{i!\left(n-i\right)!}.$$

In the 3D spatiotemporal trajectory, the order of the B-spline is set to *k* = 3 to keep the velocity and acceleration changes of the final trajectory smooth, and a higher order can be used if needed.

The initial spatiotemporal trajectory nodes are used as control points, and after fitting using the B-spline, the curve does not pass through the control points because of the B-spline curve characteristic. If left unprocessed, in some extreme cases, even if the given control point is in the safety interval, the trajectory may still collide, so the B-spline fitting margin determined in the spatiotemporal node feasibility determination step is needed.

Setting the B-spline fitting margin requires the distance information from the control point to the B-spline curve to be determined. With reference to Wang W’s research [33], in the definition of the point–curve distance, for a certain control point $Q_{i}\left(x_{qi},y_{qi},t_{qi}\right)$ in the X-Y-T three-dimensional spatiotemporal coordinate system, the nearest node $P_{i}\left(x_{pi},y_{pi},t_{pi}\right)$ can be found in the B-spline curve with the distance from the node, then the distance d from the control point to the curve can be expressed as follows:

(20) $$d=min{\;}_{1\leq i\leq n+k+1}\sqrt{\left(x_{pi}-x_{qi}\right){\;}^{2}+\left(y_{pi}-y_{qi}\right){\;}^{2}+\left(t_{pi}-t_{qi}\right){\;}^{2}}$$

If the function is used to calculate the B-spline fitting margin in real time, it will lead to excessive computation in the node expansion process, so a fixed value is preferred as the B-spline fitting margin here. According to the local variability of the B-spline curve, if only a change in a control point takes place, only local changes in the curve occur, and the other parts of the curve are not changed [34]. Thus, the third-order B-spline curve changes in a control point $Q_{i}$, the impact of the curve segments of the four control points to determine the direction of expansion is limited, and the spatial mesh step $d_{xy}$ is fixed for the exhaustive substitution validation. The maximum distance between the control points and the curve is used as the B-spline fitting margin. The subsequent optimization has node safety constraints, again to ensure that the optimization to avoid collision occurs, successfully reduce the amount of computation, and ensure the optimization of the spatial safety of the trajectory.

### 4.2. Constraint-Based Optimization

To ensure that the optimization interval is sufficient and reduces the waste of the solution space, inspired by Zhou B’s research [34], for a k-degree B-spline trajectory defined by M + 1 control points $\left[Q_{0},Q_{1},\cdots ,Q_{M}\right]$, we optimize the subset of M + 1 − 2*k* control points $\left[Q_{k},Q_{k+1},\cdots ,Q_{M-k}\right]$. The first and last *k* control points should not be changed because they determine the boundary state.

The control points $\left[Q_{k},\cdots ,Q_{M-k}\right]$ are used as variables, and the optimal control points are obtained by constructing a quadratic programming (QP) optimization problem. To ensure the uniformity of the B-spline curve, the control points $\left[Q_{k},\cdots ,Q_{M-k}\right]$ only change their spatial positions, i.e., only the *x* and *y* coordinates change within the optimization boundary values, and the t coordinate remains unchanged.

The control point optimization margin value $e_{h}$ is determined by the optimization boundary margin in the feasibility step, which determines the range of coordinate changes of the optimized control point, and the value is adjustable so that a better value can be obtained by repeating the test.

The objective function of optimization consists of multiple cost functions, and each cost function is specified first. Finally, the coefficients of each item in the cost function are empirically adjusted. The objective function is shown as follows:

(21) $$f_{total}=\lambda_{s}f_{s}+\lambda_{d}f_{d}+\lambda_{v}f_{v}$$

where the cost function consists of three terms: the smooth optimization function $f_{s}$, safety optimization function $f_{d}$, and speed optimization function $f_{v}$; $\lambda_{s},\lambda_{d},\lambda_{v}$ are the respective coefficients.

According to the hodograph property of the B-spline curve, the derivatives of each order of the curve, which is still a B-spline curve, have the same properties and can be linearly represented by the original control points. Therefore, the velocity and acceleration as the first- and second-order derivatives of the B-spline curve can be expressed linearly by the control points. The velocity $v_{i}$ and acceleration $a_{i}$ can be obtained as follows:

(22) $$\left\{\begin{array}{l} v_{i}=\frac{1}{d_{t}}\left(Q_{i+1}-Q_{i}\right),i=k,k+1,\cdot \cdot \cdot ,M-k,\\ a_{i}=\frac{1}{d_{t}}\left(v_{i+1}-v_{i}\right),i=k,k+1,\cdot \cdot \cdot ,M-k-1 \end{array}\right.$$

Referring to Zhu Z’s research [35], the smooth term uses the elastic band algorithm to improve the smoothness of the trajectory, and the control points should be uniformly distributed in a straight line. The smaller the sum of the equation, the smoother the trajectory. The specific smoothness term is expressed as follows:

(23) $$f_{s}=\sum_{i=k}^{M-k}{\left|\left|\left(Q_{i+1}-Q_{i}\right)+\left(Q_{i-1}-Q_{i}\right)\right|\right|}^{2}$$

The safety term keeps the control points as far away as possible from the nearest obstacles and is represented according to the risk field. The specific safety term is expressed as follows:

(24) $$\left\{\begin{array}{l} f_{d}=\sum_{i=k}^{M-k}\;w_{d}E_{s}\left(Q_{i}\right),\\ w_{d}=\left\{\begin{array}{cc} 1 & E_{s}\left(Q_{i}\right)\geq d_{0}\\ 0 & E_{s}\left(Q_{i}\right)<d_{0}. \end{array}\right. \end{array}\right.$$

where $E_{s}\left(Q_{i}\right)$ is the risk field value of the control point, as shown in Equation (17); $w_{d}$ is the logical indicator; and $d_{0}$ is the safety risk threshold.

The speed term is the penalty term for the deviation of the speed of the control point from the desired speed. The specific speed term is expressed as follows:

(25) $$f_{v}\;=\;\sum_{i=k}^{M-k}{\left(v_{i}-v_{goal}\right)}^{2}$$

where $v_{goal}$ is the target speed, and $v_{i}$ is the trajectory speed.

The optimization process also needs to meet certain constraints to ensure the feasibility and safety of the trajectory. The constraints are as follows: the scene boundary constraints, control point optimization margin constraints, node safety constraints, speed constraints, and acceleration constraints.

The control point scene boundary constraints are used to ensure that the control points are within the scene boundary. They are constructed as follows:

(26) $$\left.\left\{\begin{array}{l} x_{\;}^{\mathrm{lower}}\leq x_{qi}\leq x_{\;}^{\mathrm{upper}}\\ y_{\;}^{\mathrm{lower}}\leq y_{qi}\leq y_{\;}^{\mathrm{upper}}\\ t_{\;}^{\mathrm{lower}}\leq t_{qi}\leq t_{\;}^{\mathrm{upper}} \end{array}\right.\right.$$

where $x_{b}^{upper},x_{b}^{lower},y_{b}^{upper},y_{b}^{lower},t_{b}^{upper},t_{b}^{lower}$ are the upper and lower boundaries of the obstacles in the x,y,t directions, respectively. The specific experimental value depends on the specific settings in the experimental scene.

The control point optimization margin constraint is used to ensure that the *xy* coordinate of the control point changes within the optimization margin value. It is constructed as follows:

(27) $$\left.\left\{\begin{array}{l} x_{oldi}-e_{h}\leq x_{qi}\leq x_{oldi}+e_{h}\\ y_{oldi}-e_{h}\leq y_{qi}\leq y_{oldi}+e_{h} \end{array}\right.\right.$$

where $x_{oldi}$, $y_{oldi}$ are the corresponding control point *xy* coordinates in the initial spatiotemporal trajectory, and $e_{h}$ is the control point optimization margin value in the instantiation margin, which is used in Equation (10) as part of the instantiation margins. The value can be modified to adjust the scope of control point optimization. In the geometric range of this experiment, $e_{h}=1.5\;m$ is generally taken.

The velocity constraints and acceleration constraints are used to ensure that the motion of the robot is feasible. Considering the dynamics of the robot, the velocity constraints and acceleration constraints of the trajectory points are constructed as follows:

(28) $$\left\{\begin{array}{l} v_{min}\leq v_{i}\leq v_{max},\\ a_{min}\leq a_{i}\leq a_{max}, \end{array}\right.$$

where $v_{min}$ and $v_{max}$ are the minimum and maximum values of velocity, and $a_{min},a_{max}$ are the minimum and maximum values of acceleration, respectively. The specific experimental value depends on the specific settings in the experimental scene.

The node safety constraints are used to ensure that the final trajectory curve meets the safety constraints, and they are constructed as follows:

(29) $$\left.\left\{\begin{array}{l} x_{b}^{lower}+w_{hx}e_{v}+e_{tx}\leq x_{pi}\leq x_{b}^{upper}-w_{hx}e_{v}-e_{tx}\\ y_{b}^{lower}+w_{hy}e_{v}+e_{ty}\leq y_{pi}\leq y_{b}^{upper}-w_{hy}e_{v}-e_{ty} \end{array}\right.\right.$$

where $x_{b}^{upper},x_{b}^{lower},y_{b}^{upper},x_{b}^{lower},t_{b}^{upper}$, and $t_{b}^{lower}$ are the upper and lower boundaries of the obstacle in the *x*, *y*, *t* directions; the specific experimental value depends on the specific settings in the experimental scene; and $x_{pi}$, $y_{pi}$ are the coordinates of the nodes corresponding to the control points on the constructed curve, respectively.

Since the node safety constraints are the only constraints for nodes, rather than control points, it is necessary to introduce the corresponding optimization adjustment strategy. When the node safety constraints are not satisfied at some nodes, the optimization algorithm adopts the strategy of gradually adjusting the control points to achieve constraint satisfaction, and its adjustment direction $\theta_{adj}$ and adjustment step size $\lambda_{adj}$ are shown as follows:

(30) $$\left.\theta_{adj}=\right.\arctan\left(\frac{y_{wri}-u_{y}}{x_{wri}-u_{x}}\right)\;\lambda_{adj}=w_{adj}\sqrt{{\left(x_{qi}-x_{pi}\right)}^{2}+{\left(y_{qi}-y_{pi}\right)}^{2}}$$

where $u_{x},u_{y}$ are the coordinates of the center point of the nearest obstacle; $x_{wri}$, $y_{wri}$ are the coordinates of the control point when the node safety constraints are not met; and $w_{adj}$ is the weight of the adjustment step.

The specific adjustment of the control point *xy* coordinates is shown as follows:

(31) $$\left\{\begin{array}{l} x_{qi}=x_{oldqi}+\lambda_{adj}\cos\left(\theta_{adj}\right)\\ y_{qi}=y_{oldqi}+\lambda_{adj}\sin\left(\theta_{adj}\right) \end{array}\right.$$

**Figure 8:**
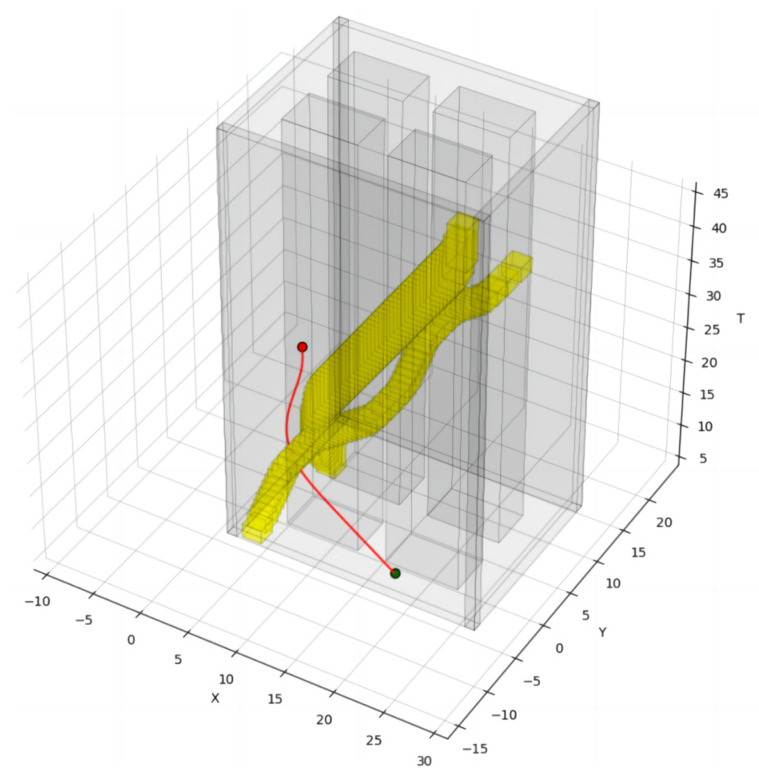
The final spatiotemporal trajectory of the incremental robot.

Finally, the smooth distribution of control points without collision and conforming to the motion constraints can be obtained through secondary optimization, and the final incremental spatiotemporal trajectory of the robot is obtained after fitting as shown in Figure 8. The red curve is the final smooth trajectory.

## 5. Simulation and Discussion

To fully verify the effectiveness and superiority of the spatiotemporal variable-step A* algorithm planning method proposed in this paper, complex motion scenarios were used for simulation. The simulations were conducted in Python using the OSQP solver. All experiments were performed on a quad-core 3.20 GHz Intel i5-6500 processor.

The simulation and comparison experiments were conducted in two distinct settings.

The first group was carried out in a small-scale scenario and compared with the spatiotemporal decoupling planning algorithm CL-CBS [7]. The aim was to assess the STP-STVS-A*’s performance in incremental robot trajectory planning within a small passing space.

The second group was conducted in a larger-scale scenario, comparing the algorithm against ETPMR, the state-of-the-art spatiotemporal joint planning algorithm [36]. This comparison aimed to highlight the differences between this method and other spatiotemporal joint planning approaches.

### 5.1. Small-Scale Scenario Simulation

In the first simulation process, we selected a field-shaped, complex motion scene containing multiple motion conflict points. This scene covers horizontal and vertical conflicts between multiple robots and can be effectively used to test the planning ability of the spatiotemporally variable-step-size A* algorithm in complex situations.

Due to the complexity and large span of the motion scene, time domain T is set to 70 s, and the predicted length of obstacle trajectories of the existing robots and the predicted length of static obstacles, Tp, should be consistent with the planning time domain, so Tp = 70 s. The other relevant parameters of the experiment are given in Table 1.

The experimental scene contains four existing robots that have already run in the scene and one introduced incremental robot. The parameters of each robot are given in Table 2.

**Figure 9:**
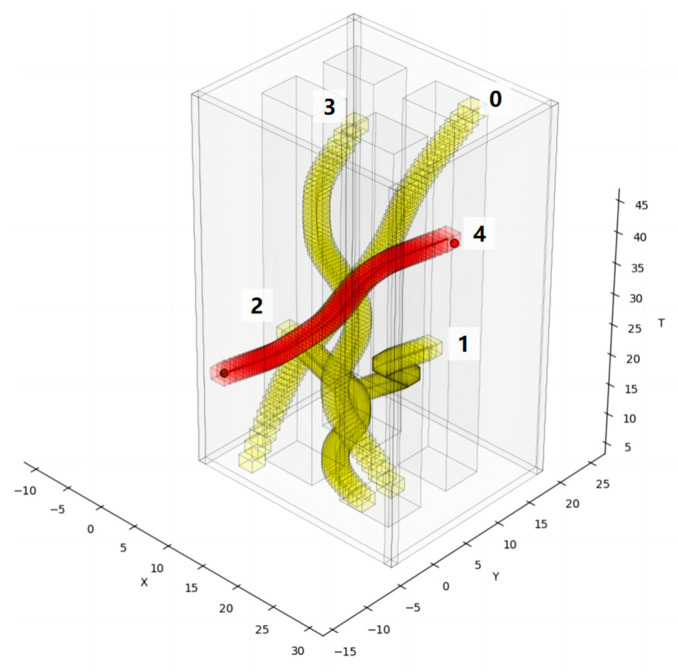
Result diagram of STP-STVS-A*.

As shown in Figure 9, in the setup scene, there are already four existing robots running in the scene. The yellow obstacles in the figure indicate the spatiotemporal occupation state of the existing robots, the red obstacles indicate the spatiotemporal occupation state of the incremental robot, and the red curves indicate the spatiotemporal trajectories of the incremental robot. The incremental robot starts running from the 15th second in the positive direction of the x-axis at a speed of 1 m/s. To avoid colliding with the running, pre-existing intelligent robot 3, incremental robot 4 delays its turning time, ensures a safe traveling space, and arrives at the target position in a manner that is close to the shortest traveling trajectory without affecting the operation of other pre-existing intelligent robots.

**Figure 10:**
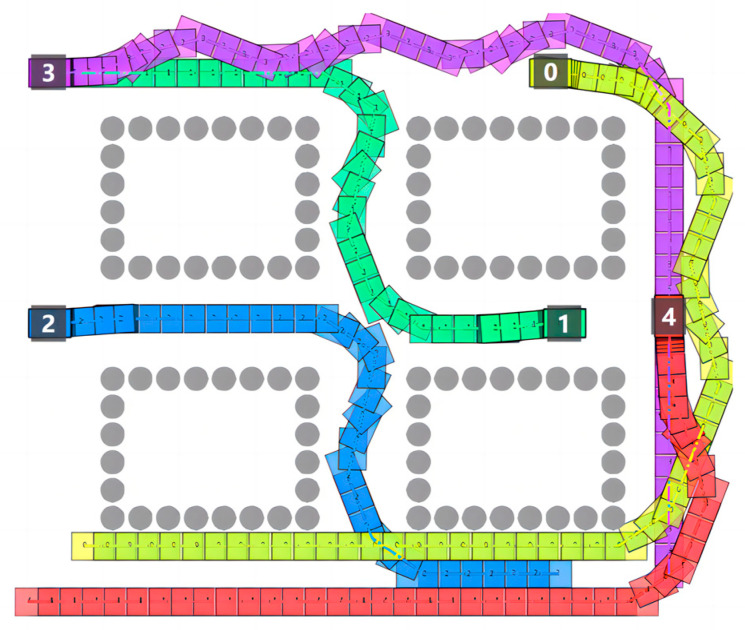
Result diagram of CL-CBS.

For comparison and discussion, the CL-CBS in Wen L’s research [7] was chosen to conduct simulation experiments in the same scene. The experimental results of the comparison with the CL-CBS are shown in Figure 10. Since the running speed of each robot cannot be set individually in the CL-CBS, all the robots can only run at a uniform speed of 1 m/s.

**Figure 11:**
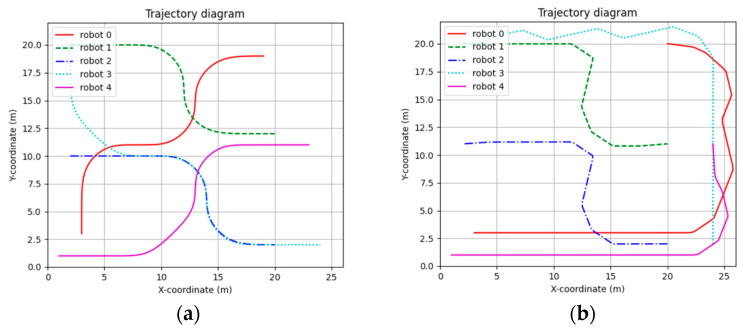
Diagram of trajectory: (**a**) STP-STVS-A* and (**b**) CL-CBS.

Figure 11 shows the trajectory of the STP-STVS-A* and the CL-CBS. It can be seen that the trajectory of the method proposed in this paper is smoother and closer to the shortest traveling trajectory compared to the CL-CBS. This is due to the spatiotemporal decoupling planning used in the CL-CBS, which generates the final trajectory by calculating the speed and the path result separately. The calculation of this method is also more conservative in a complex, dynamic environment, which can easily lead to a suboptimal trajectory. In addition, the trajectory results of the CL-CBS for a wheeled mobile robot are too tortuous to be used.

Moreover, the CL-CBS cannot be used to plan the trajectories of incremental robots without affecting the existing running robots, so there is obvious interference with the trajectories of robot 1 and robot 2. In contrast, the method proposed in this paper can be used for trajectory computation of incremental robots and can repeatedly iterate incremental robots without affecting the existing running robots, making it more practical in some scenarios.

**Figure 12:**
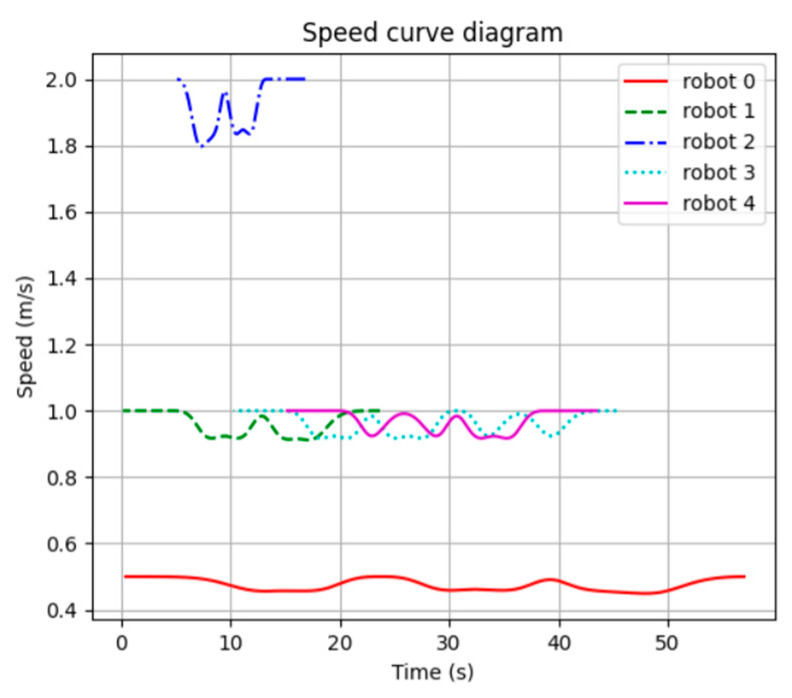
Speed curve diagram of STP-STVS-A*.

Figure 12 shows the speed curve of each robot constructed using the results of this method. It can be seen that the speed of each robot is basically kept at the target speed for movement.

The speed error rate was introduced as an index to better illustrate the speed smoothness and speed fluctuation. The speed error rate can be expressed as follows:

(32) $$E_{vri}=\left|v_{ri}-v_{goal}\right|/v_{goal}\times 100\%$$

where $v_{ri}$ is the current speed of the robot in the result of the algorithm, and $v_{goal}$ is the target speed of the robot.

**Figure 13:**
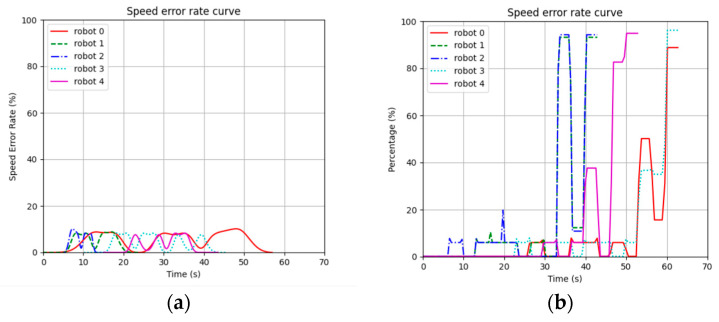
Plot of speed error rate curve: (**a**) STP-STVS-A* and (**b**) CL-CBS.

Figure 13 shows the curve of the speed error rate for each robot in the results of the STP-STVS-A* and the CL-CBS.

**Figure 14:**
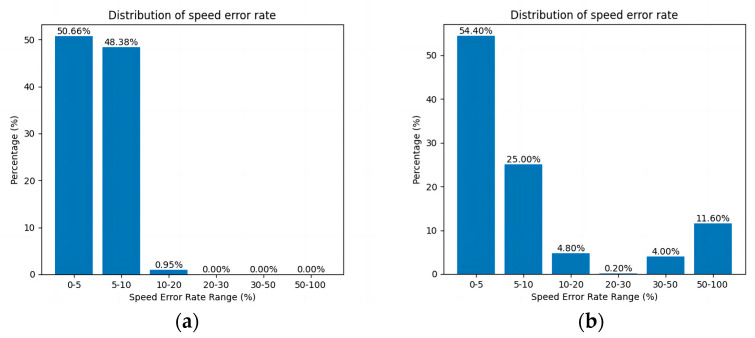
Distribution of speed error rate: (**a**) STP-STVS-A* and (**b**) CL-CBS.

Figure 14 shows the distribution of the speed error rate for each robot in the results of the STP-STVS-A* and the CL-CBS.

It can be seen that the speed of the robot in the CL-CBS varies more drastically, which is as attributed to the lack of optimization and instantiation steps, so there is no smoothing of the speed. The speed of the robot planned using this method changes smoothly and continuously and rarely changes drastically, and the difference with the target speed is basically no more than 10%, which ensures that the robot is more capable of running in the set target state. In particular, the computational target, i.e., incremental robot 4, has a stable change of speed and smooth and efficient movement.

Table 3 shows the comparison of the distance traveled by each robot for the two methods. It can be seen that the trajectory of the STP-STVS-A* proposed in this paper is shorter after the spatiotemporal trajectory instantiation optimization compared to the pre-optimization trajectory, and the instantiation processing can make the resultant trajectory smoother and more efficient. Compared with the CL-CBS, the trajectory of the STP-STVS-A* proposed in this paper has a shorter travel length and is closer to the shortest driving trajectory. Because the method proposed in this paper utilizes the trajectory search method with a variable time step, each robot can run at different target speeds, which is more practical in some scenarios.

### 5.2. Large-Scale Scenario Simulation

To further corroborate the effectiveness of this method, an additional simulation in a larger-scale scenario is conducted, comparing it with other methods as detailed below.

In contrast to the first simulation experiments, a larger-scale scenario with increased size and static obstacles was employed. This setting allows for a comparison of the method’s effectiveness in handling incremental robots amidst a larger passable area.

Given the expanded scope of the motion scene, a larger time domain is required. Accordingly, the time domain (T) is set to 140 s, aligning with both the predicted length of obstacle trajectories for existing robots and the predicted length of static obstacles (Tp), also set to 140 s. The size of the robot remains consistent with that of the initial experiment, while the scene size is enlarged. Detailed specifications are provided in Table 4.

The experimental scene contains seven existing robots that have already run in the scene and one introduced incremental robot. The parameters of each robot are given in Table 5.

**Figure 15:**
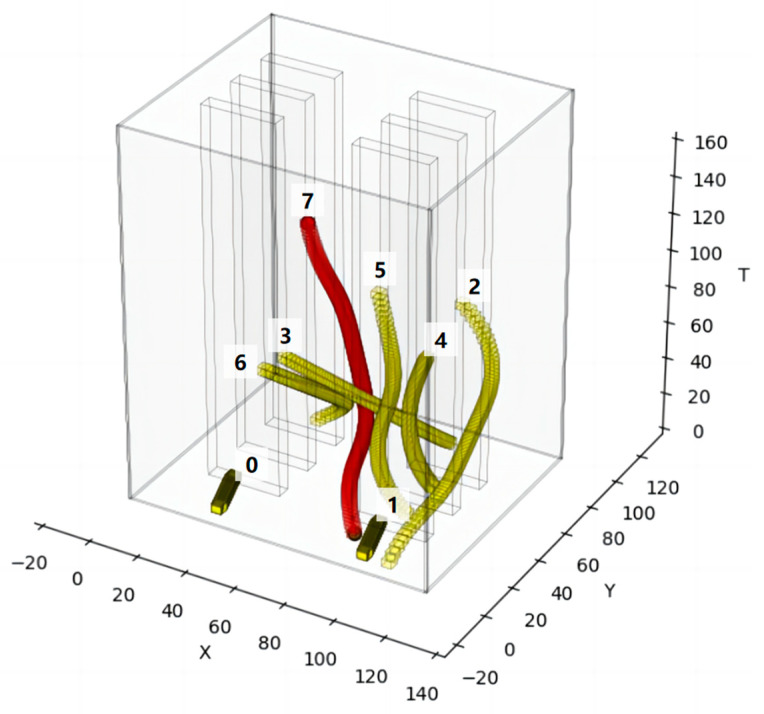
Result diagram of STP-STVS-A*.

As depicted in Figure 15, the scene is set with seven existing robots already in motion. Yellow obstacles represent the spatiotemporal occupancy of these existing robots, while red obstacles indicate the spatiotemporal occupancy of the incremental robot. Incremental robot 7 commences its motion at a speed of 1 m/s along the positive x-axis direction starting from the 20th second. To avoid collision with the moving intelligent robot 5, incremental robot 7 veers left, endeavoring to maintain its speed while ensuring a safe driving space. It reaches the target position following a trajectory closely resembling the shortest driving path, without disrupting the operation of other existing intelligent robots.

**Figure 16:**
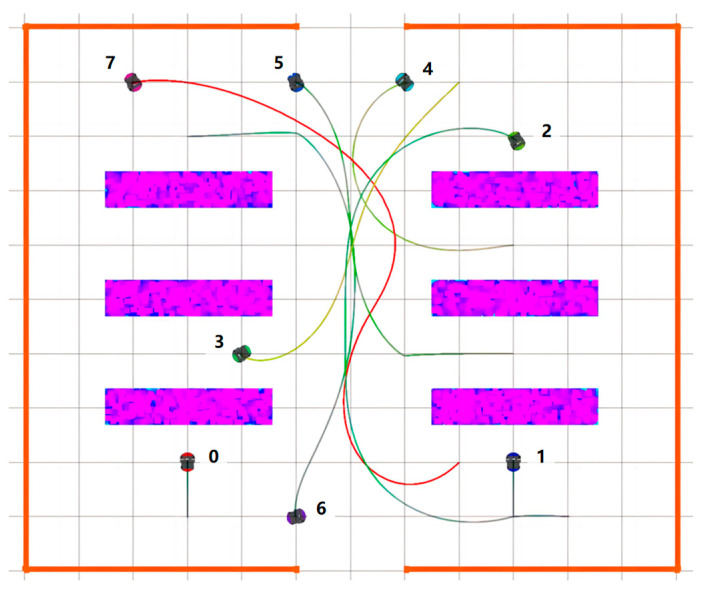
Result diagram of ETPMR.

For comparison, simulation experiments were conducted using the Efficient Trajectory Planning for Multiple Non-holonomic Mobile Robots (ETPMR) from Li J’s research [36]. This method utilizes spatiotemporal joint planning and a graph-based multi-agent path planner. The results, shown in Figure 16, were standardized to a uniform speed of 1 m/s for all robots. The method does not use a spatiotemporal grid map to display the results, so the planar graph output by its original program is used in Figure 16.

**Figure 17:**
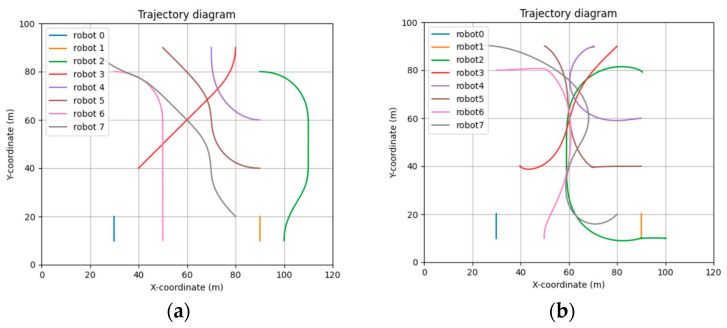
Diagram of trajectory: (**a**) STP-STVS-A* and (**b**) ETPMR.

Figure 17 shows the trajectories generated by the STP-STVS-A* and the ETPMR. Notably, the trajectory produced by the STP-STVS-A* is visibly shorter and more direct. This discrepancy arises from the priority grouping mechanism employed in the ETPMR, which results in certain robot outcomes being constrained by priority, introducing a distinct disruptive factor not present in our approach. Furthermore, in scenarios with ample passable space, the ETPMR tends to adopt a more conservative approach. The majority of robot trajectories converge within the central channel, leaving more free space on either side, potentially leading to suboptimal trajectories. Nonetheless, in comparison to the CL-CBS, the ETPMR yields smoother trajectories and is suitable for application in wheeled mobile robots.

Similar to traditional planning methods, the ETPMR encounters challenges in planning the trajectory of incremental robots without impacting the motion of existing operating robots, resulting in notable interference with the trajectories of robot 3 and robot 5. In contrast, the method proposed in this paper facilitates trajectory calculation for incremental robots without disrupting the motion of existing operating robots. This capability allows for the iterative planning of incremental robots without affecting ongoing operations, rendering it more practical in certain scenarios.

**Figure 18:**
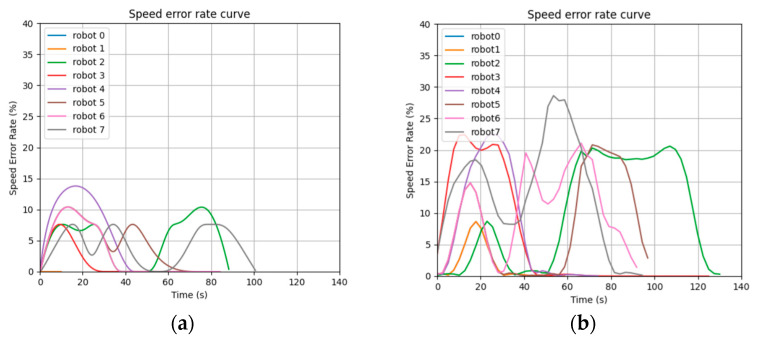
Plot of speed error rate curve: (**a**) STP-STVS-A* and (**b**) ETPMR.

**Figure 19:**
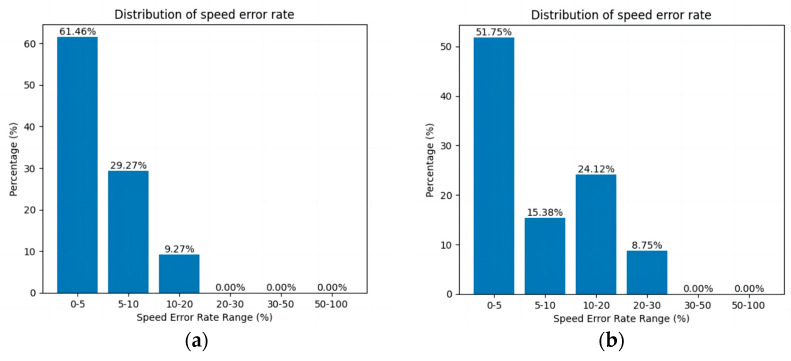
Distribution of speed error rate: (**a**) STP-STVS-A* and (**b**) ETPMR.

The representation is the same as that of the first simulation experiment. Figure 18 shows the curve of the speed error rate for each robot in the results of the STP-STVS-A* and the ETPMR. Figure 19 shows the distribution of the speed error rate for each robot in the results of the STP-STVS-A* and the ETPMR.

It is evident that the STP-STVS-A* exhibits a relatively low error rate, with differences from the target speed typically remaining below 20%. This is particularly notable for incremental robot 7, where the STP-STVS-A* excels. In contrast, the space segmentation approach utilized in the ETPMR impacts the passable space, leading to increased velocity fluctuations.

Table 6 provides a comparison of the distance traveled by each robot under the two methods. As observed in the first experiment, trajectories are shorter after spatiotemporal trajectory instantiation optimization. Furthermore, compared to the ETPMR, trajectories generated by the STP-STVS-A* are shorter and closer to the shortest driving path. This is attributed to the STP-STVS-A*’s utilization of a trajectory search approach with variable time steps, enabling each robot to operate at different target speeds—a feature that enhances practicality in certain scenarios.

In summary, the ETPMR exhibits strong performance in scenarios involving multi-agent-coordinated motion planning. However, our tests indicate that this method struggles when applied to incremental robot trajectory calculation scenarios. In these scenarios, an incremental robot must enter the operational environment without disrupting the ongoing activities of existing robots.

Priority grouping planning can uniformly calculate the initial guidance trajectories for all agents and partition feasible corridors, thereby improving computational efficiency. However, due to the solution method of space-time corridors [37], the solution space is entirely partitioned, preventing the introduction of new solution spaces. If an incremental agent is reintroduced for calculation, the trajectory results of other agent sets will be affected, and the calculation results cannot be iterated.

This paper does not address the incremental robot problem, which requires iteration based on the order in which robots enter the scene. Furthermore, this method requires that multiple agents start simultaneously, rendering it unsuitable for different startup situations in the robot increment problem.

Therefore, the STP-STVS-A* is more aptly suited for scenarios involving incremental robot trajectory planning.

### 5.3. Algorithm Performance Analysis

Through experimentation in different scaled scenarios, the STP-STVS-A*’s efficacy across different solution spaces has been verified. When compared with two state-of-the-art methods, CL-CBS and ETPMR, it demonstrates good algorithmic performance and a unique problem-solving capability in the context of incremental robot trajectory planning.

In order to show the functional comparison between methods more concisely, the comparison between methods is tabulated. Refer to the figures and tables in Section 5.1 and Section 5.2 for specific data.

As illustrated in Table 7, the method proposed in this paper excels in both path smoothing and velocity smoothing, yielding results that closely approximate the shortest path. It allows for different target speeds to be set for each robot, and incremental robots that depart at different times can be iteratively calculated. Importantly, robots that depart later do not interfere with the movement of existing robots. These performance results underscore the spatiotemporal variable step size A* algorithm’s exceptional effectiveness in ensuring the safe trajectory planning of incremental robots in complex scenarios.

However, a notable drawback of this method is its computational efficiency. In small-scale simulations, the algorithm’s computation time is 2.02 s, compared to 0.12 s for the CL-CBS. In large-scale scenario simulations, the computation time of the STP-STVS-A* increases to 4.20 s, while the ETPMR algorithm’s computation time is 0.62 s. The reasons for the long calculation time of this method are as follows:

To ensure the safety of robot motion, the algorithm increases the partial collision decision margin, and the introduction of a risk field also augments the computational load. Furthermore, to ensure iterability, part of the collision determination of the existing robot will be repeated. In aspects other than technical methods, the algorithm is written in Python, while the CL-CBS and ETPMRs are written in C. If the algorithm was converted into C, it is anticipated that the computation time could be reduced several-fold, thereby narrowing the time gap. However, in the problem addressed in this paper, the incremental agent trajectory is considered as global planning; the primary concern is the excellence of the resulting trajectory’s performance. There is less need for immediacy in this problem.

The method proposed in this paper is computed in a three-dimensional spatiotemporal setting, which leads to a solution space with a more complete trajectory. The initial spatiotemporal trajectory search using the spatiotemporal variable-step-size A* algorithm effectively takes into account safety while enhancing the flexibility of the solution so that the behavioral performance is more flexible, the results are more reasonable, and the trajectory planning for incremental robots can be carried out without affecting the existing robots.

### 5.4. Discussion

The method proposed in this paper holds significant promise for solving the safe trajectory planning challenges of incremental wheeled mobile robots. It has potential applications in various fields, such as intelligent networked vehicles and storage and transportation robots.

For instance, in intelligent networked vehicles, where the iterative calculation of trajectories is crucial, this method can be utilized to determine spatiotemporal trajectories and devise macro-control strategies for safe and efficient traffic flow. Similarly, in scenarios involving incremental robots like those in storage and transportation, this method can optimize robot movement trajectories to enhance transportation efficiency within warehouses.

While our approach demonstrates promising results in simulation tests, it may encounter challenges in real-world scenarios due to factors like sensor errors, environmental fluctuations, and external disturbances. Despite incorporating constraints like speed control error margins, due to the error between the robot position and the calculated trajectory in practical application, the error may accumulate after multiple iterations of different robots. Moreover, it is difficult to control the timing of multiple robots entering the scene with absolute accuracy, and the accumulation of errors cannot be ignored. Therefore, it is necessary to further study how to reduce the impact of errors.

In conclusion, while the proposed path planning method carries both theoretical and practical significance, it faces challenges and limitations in real-world application. Further research and refinement are necessary to effectively apply this method in practical scenarios.

## 6. Conclusions

This paper proposes an incremental robot trajectory planning method based on the spatiotemporal variable-step-size A* algorithm to address the problem of safe trajectory planning for incremental robots in multi-intelligent complex scenarios. After constructing the known conditions, the initial spatiotemporal trajectory computation of the target speed can be set using the spatiotemporal variable-step-size A* algorithm, which ensures that the search is completed at the target speed based on the variable time step and ensures that the initial spatiotemporal of the robot is safe, and there is room for optimization through the node feasibility determination. Then, by using the obtained initial spatiotemporal trajectory nodes as control points, fitting with B-spline curves, and numerical optimization, the trajectory instantiation is realized, and the final smooth spatiotemporal trajectory is generated.

Simulation results show that the proposed spatiotemporal joint planning method has smoother trajectory paths and velocities, making it more suitable for wheeled mobile robots. Different target velocities can also be set to iteratively compute incremental robots without affecting the motion of existing robots, which makes this method suitable for trajectory computation in complex motion scenarios and more flexible than the traditional planning methods. The planning results also show increased safety and efficiency.

In future work, we intend to validate our algorithm through experimental analysis using actual robots. This will involve testing the algorithm’s performance under various practical conditions. By conducting these experiments, we aim to further demonstrate the practical applicability and effectiveness of our method in real-world scenarios. Additionally, we plan to incorporate more complex spatiotemporal constraints into the planning process and model the inconsistency between different robots in the cost function, which will aid adaptation to more complex motion scenarios in multi-agent robotic systems.

## Figures and Tables

**Table 1:** Parameters of a simulation experiment.

Parameter	Value
Length of robot Lv/m	5
Width of robot Wv/m	3
Intelligent robot speed range v/(m s^−1^)	[0, 2]
Total scene length L/m	26
Total scene width W/m	22

**Table 2:** Parameters of simulation robots.

Parameter	Existing Robot 0	Existing Robot 1	Existing Robot 2	Existing Robot 3	Incremental Robot 4
Start position (X0,Y0)/m	(3,3)	(2,20)	(20,2)	(24,2)	(1,1)
Target position (Xg,Yg)/m	(20,20)	(20,11)	(1,11)	(2,20)	(24,11)
Target speed Vs/(m s^−1^)	0.5	1	2	1	1
Departure time Ts/s	0	0	5	10	15

**Table 3:** Method distance comparison.

Group	STP-STVS-A* before Optimization (m)	STP-STVS-A* after Optimization (m)	CL-CBS (m)
Robot 0	28.48	27.08	41.33
Robot 1	23.65	22.76	25.87
Robot 2	23.65	22.71	25.85
Robot 3	35.31	34.02	40.73
Robot 4	28.48	27.65	32.96
Total	124.28	115.58	166.58

**Table 4:** Parameters of the second simulation.

Parameter	Value
Total scene length L/m	100
Total scene width W/m	120

**Table 5:** Parameters of second simulation’s robots.

Parameter	Existing Robot 0	Existing Robot 1	Existing Robot 2	Existing Robot 3	Existing Robot 4	Existing Robot 5	Existing Robot 6	Incremental Robot 7
Start position (X0,Y0)/m	(30,10)	(90,10)	(100,10)	(80,90)	(90,60)	(90,40)	(30,80)	(80,20)
Target position (Xg,Yg)/m	(30,20)	(90,20)	(90,80)	(40,40)	(70,90)	(50,90)	(50,10)	(20,90)
Target speed Vs/(m-s-1)	1	1	1	1	1	1	1	1
Departure time Ts/s	0	0	0	0	0	5	10	20

**Table 6:** Method distance comparison.

Group	STP-STVS-A* before Optimization (m)	STP-STVS-A* after Optimization (m)	ETPMR (m)
Robot 0	10.00	10.00	10.00
Robot 1	10.00	10.00	10.19
Robot 2	88.28	83.73	127.82
Robot 3	66.56	65.45	70.33
Robot 4	44.14	40.28	55.42
Robot 5	72.42	68.70	75.70
Robot 6	84.14	81.66	91.06
Robot 7	100.71	96.53	112.57
Total	476.25	456.35	553.09

**Table 7:** Comparison of method results.

Project	STP-STVS-A*	CL-CBS	ETPMR
Path smoothness	Path smoothest	Path zigzagging	Path smooth
Result length	Shortest, closest to optimal path	Longer	Shorter
Speed smoothness	Smooth speed change, small difference from the target speed	Dramatic speed change, large difference from the set speed	Smoother speed changes
Target speed configurability	Different target speeds can be set	Only has one set speed	The speed varies within different groups
Incremental robot iterability	Incremental robots can be iterated starting at different times	Robots start at the same time and cannot be iterated	Cannot be iterated
Algorithm computation time	Longe	Shortest	Short

## Data Availability

Data are contained within the article.
